# Sodium Homeostasis, a Balance Necessary for Life

**DOI:** 10.3390/nu15020395

**Published:** 2023-01-12

**Authors:** Antonio Bernal, María A. Zafra, María J. Simón, Javier Mahía

**Affiliations:** Department of Psychobiology and Mind, Brain and Behavior Research Center (CIMCYC), University of Granada, Campus de Cartuja, 18071 Granada, Spain

**Keywords:** sodium homeostasis, hypernatremia, hyponatremia, taste, salt intake, kidney, natriuresis, excitatory and inhibitory circuits, posterior hypothalamus

## Abstract

Body sodium (Na) levels must be maintained within a narrow range for the correct functioning of the organism (Na homeostasis). Na disorders include not only elevated levels of this solute (hypernatremia), as in diabetes insipidus, but also reduced levels (hyponatremia), as in cerebral salt wasting syndrome. The balance in body Na levels therefore requires a delicate equilibrium to be maintained between the ingestion and excretion of Na. Salt (NaCl) intake is processed by receptors in the tongue and digestive system, which transmit the information to the nucleus of the solitary tract via a neural pathway (chorda tympani/vagus nerves) and to circumventricular organs, including the subfornical organ and area postrema, via a humoral pathway (blood/cerebrospinal fluid). Circuits are formed that stimulate or inhibit homeostatic Na intake involving participation of the parabrachial nucleus, pre-locus coeruleus, medial tuberomammillary nuclei, median eminence, paraventricular and supraoptic nuclei, and other structures with reward properties such as the bed nucleus of the stria terminalis, central amygdala, and ventral tegmental area. Finally, the kidney uses neural signals (e.g., renal sympathetic nerves) and vascular (e.g., renal perfusion pressure) and humoral (e.g., renin–angiotensin–aldosterone system, cardiac natriuretic peptides, antidiuretic hormone, and oxytocin) factors to promote Na excretion or retention and thereby maintain extracellular fluid volume. All these intake and excretion processes are modulated by chemical messengers, many of which (e.g., aldosterone, angiotensin II, and oxytocin) have effects that are coordinated at peripheral and central level to ensure Na homeostasis.

## 1. Introduction

Sodium (Na) is frequently considered antagonistic to health, given that a high-salt (NaCl) diet is harmful. However, the appetite for NaCl appears to be an innate mechanism, and an elevated NaCl intake may serve to protect against dehydration [1,2,3]. Maintenance of this ion within appropriate levels (Na balance) is not only an adaptive process necessary for survival but also an essential component of hydromineral homeostasis (Na and fluid balances).

Na is the primary cation in extracellular fluid (ECF) and, along with associated anions, constitutes 90% of ECF osmolality [4,5]. ECF, which contains slightly more than one-third of total body fluid, has a Na concentration of around 144 mOsm/L. Almost all the remaining body fluid is contained in intracellular fluid (ICF), which has a lower concentration of Na (10 mOsm/L) [6]. Given that the maintenance of these water and Na levels is essential for adequate cell functioning and because the cell membrane has low permeability to solutes, water moves by osmosis from areas of lower to higher solute concentration to equalize osmotic ECF and ICF concentrations [7,8]. Hence, Na is essential to maintain electrolyte and water balances (hydromineral regulation).

The Na balance is the difference between the amount of Na absorbed by the gut and the amount excreted via urine, feces, and skin [4,8]. This review does not address other functions associated with this cation, such as the generation of nerve impulses, heart activity, and some metabolic functions.

**Figure 1:**
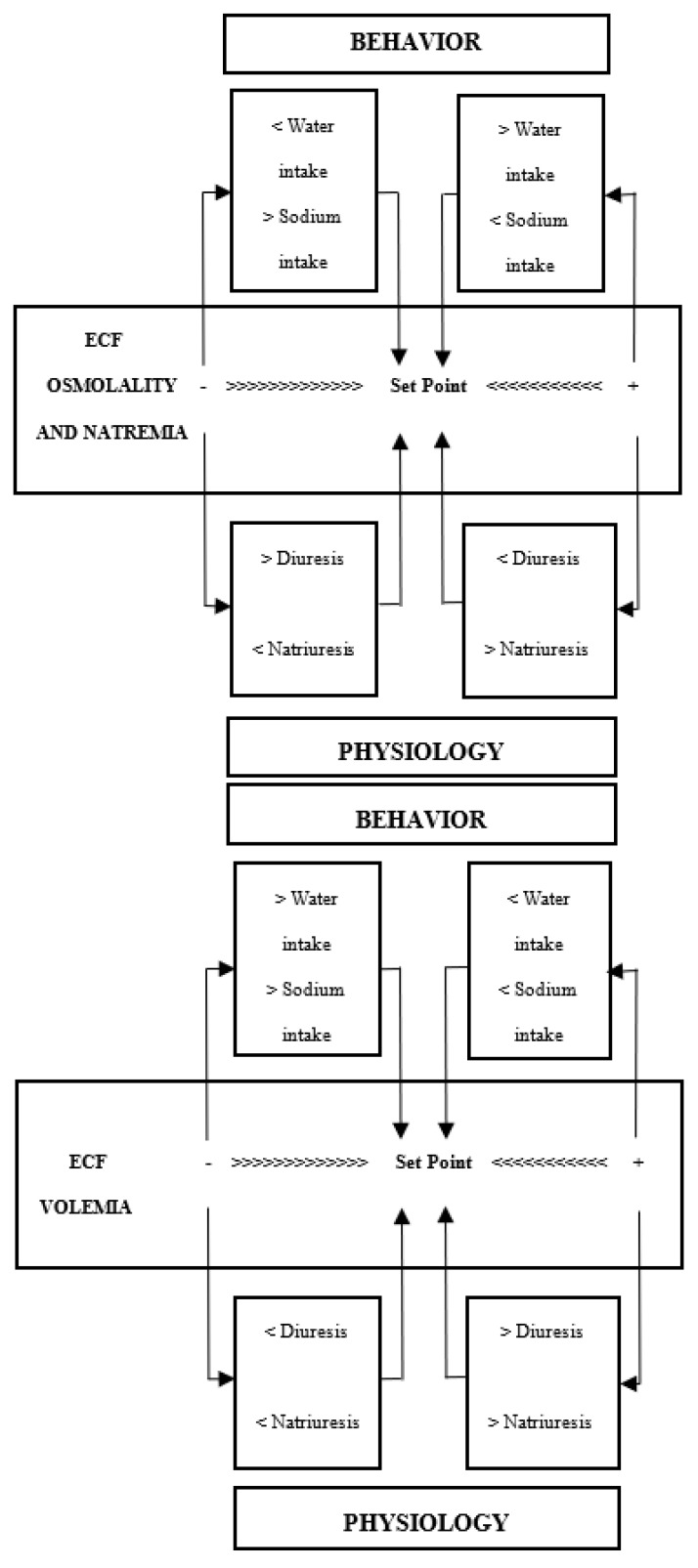
Behavioral and physiological mechanisms to maintain the osmolality/natremia (**upper**) and volemia (**lower**) of extracellular fluid (ECF) (from Mahía and Bernal [7], with permission from Elsevier).

Two biological mechanisms can alter the Na balance and hydromineral homeostasis [7] (see Figure 1). The first is largely determined by the intake of NaCl, which accumulates in the extracellular space and leads to the displacement of water from cell to extracellular space, producing cell dehydration. In this situation, the appetite for Na is inhibited and its excretion is increased (natriuresis), while the intake of water is enhanced and the excretion of fluid is reduced (antidiuresis). Conversely, a reduction in ECF osmolality/natremia leads to the inhibition of water intake and promotion of diuresis, increasing the appetite for Na and suppressing natriuresis (Figure 1, upper). The second mechanism results from the loss of intravascular fluid (hypovolemia), one of the components of ECF (Figure 1, lower). Diarrhea, vomiting, hemorrhages, sweating, renal disease, and cardiovascular disorders are sometimes accompanied by the loss of intravascular volume. Hypovolemia triggers compensatory behavioral and physiological reactions to conserve Na and body fluids. These include a thirst and appetite for Na, especially for isotonic drinks containing the same sodium chloride concentration as intravascular fluid (0.15 M), as well as antidiuretic and antinatriuretic responses to retain water and Na. Conversely, ECF hypervolemia reduces the intake of water and Na and increases diuresis and natriuresis.

This review first addresses the NaCl intake process (behavioral mechanism) and the neural and humoral pathways that transmit information to the central nervous system (CNS). Next, it examines the brain circuits that monitor the Na balance and stimulate or inhibit NaCl intake. Finally, the process of renal Na excretion is described (physiological mechanism).

## 2. Behavioral Mechanism: Salt Intake

The Na ion [Na^+^] is an essential micronutrient that cannot be generated by endogenous processes and must be obtained through the diet [9]. The need to maintain an appropriate Na balance has led living organisms to develop mechanisms that identify the presence of Na the instant it enters the body [10]. The first level of detection is located in the oral cavity, from where it accesses the digestive system and is absorbed in the blood.

### 2.1. Detection and Processing in the Oral Cavity

Na is detected by the taste system within the oral cavity. The mineral generates a salty taste that guides and maintains its consumption [11,12,13,14]. The gustatory stimulus used as prototype to study the perception of this taste was table salt (NaCl), physiologically the most important salt in the diet, which induces the purest salty taste [11,15], and biologically the most relevant to maintain biochemical balance [16].

**Figure 2:**
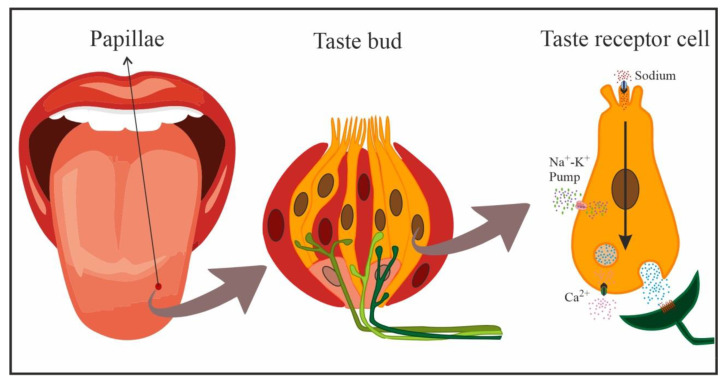
Schematic representation of cells in a taste bud, and transduction of NaCl taste in a taste receptor cell (yellow: taste receptor cells, red: support cells, pink: immature precursor cells). Taste receptor cells form synapses with afferent nerve fibers that penetrate the taste bud, crossing the basal membrane.

In mammals, gustatory receptors are observed throughout the oral cavity, especially on the tongue, where they are located on protuberances designated papillae. There are four types of papillae according to their shape and localization: fungiform (anterior two-thirds of the tongue), foliate (side toward the back), circumvallate (on the back), and filiform (anterior two-thirds of the tongue). Cells that detect NaCl are mainly located in fungiform papillae [12,17]. In general, gustatory papillae can contain several hundreds of taste buds, onion-shaped organs (Figure 2) that house taste receptor cells (50–100 cells each), i.e., the cells responsible for detecting and transducing taste stimuli (transforming chemical signals into neural activity). In the taste bud, these cells are mechanically held together by tight junctions that separate apical areas from the remaining basolateral region, forming an almost impermeable barrier. Only the apical membrane above the tight junctions (2–3% of the total membrane area of the cell) is exposed to saliva, while the basolateral area remains in a relatively constant interstitial milieu [12,13,14,18].

Taste receptor cells are bipolar neuroepithelial cells with finger-like projections (microvilli) on their apical end that project towards the oral cavity. These microvillar processes protrude into the oral milieu through a small opening, the bud pore, in order to sample the chemical environment. When salty stimuli dissolved in saliva come into contact with these microvilli, the taste cells generate a biological signal that is transmitted to afferent neural fibers that penetrate the base of taste buds and extensively ramify. These afferent nerve fibers form synapses with taste receptor cells and respond to excitatory neurotransmitters released by the latter [19], relaying this information to the brain to code the taste sensation [10,14].

There appear to be two types of salty taste receptor cells in the lingual epithelium. One of them, specialized in coding the perceptual quality of “salty” taste, is highly selective for Na, being activated by low–medium concentrations of NaCl and blocked by lingual application of amiloride (a diuretic drug). The other type is activated by high (aversive) concentrations of NaCl (>150 mM), is amiloride-insensitive, and is non-selective for Na (other salts are equally effective). The latter type of taste receptor cells appears to be innervated by neural pathways associated more with aversion than with taste coding [12,14,19,20].

Taste reception begins when chemicals interact with taste receptors located at the apical segment of the taste cells. The receptors that mediate the transduction of salty taste are ion channels [12,19], designated epithelial Na channels (ENaCs), which are specialized in the detection or recognition of sodium chloride [11,12,15,16,19]. Interestingly, and as expected, it has been demonstrated that the gustatory response to Na in deficient animals can be augmented by some of the hormones involved in its regulation, e.g., aldosterone or angiotensin II (Ang II), through an increase in the number of active amiloride-sensitive Na channels in the apical membrane of taste cells [9,21,22].

With the increase in salivary Na concentration, this cation directly enters cells through amiloride-sensitive ENaCs on the apical membrane, following electrochemical gradients. The increase in positive charges inside the taste receptor cell creates a depolarization of the cell membrane that generates action potentials passively conducted towards the basolateral region of the taste receptor cell in a dose-dependent manner, i.e., the action potential frequency is dependent on the stimulus intensity. Here, the opening of voltage-activated Ca^2+^ gives rise to the influx of Ca^2+^ ions and the release of the corresponding neurotransmitter onto postsynaptic gustatory afferent fibers (Figure 2) that carry the taste signal to higher-order systems [10,16]. Detection by the nonspecific amiloride-insensitive mechanism is less well known and appears to take place below the apical membrane and taste cell tight junctions (basolateral detection) [13,16].

NaCl taste afferents are the peripheral processes of pseudounipolar neurons located in geniculate cranial ganglia. The chorda tympani nerve, with cell bodies in the geniculate ganglion, appears to be mainly responsible for transmitting information on the presence of Na in the oral cavity to the brain [18,23,24]. Thus, bilateral transection of this nerve severely disrupts the ability to discriminate Na from other salts [25]. In humans, the salty taste is generated at concentrations above 40 mM, a very similar threshold to that in rats (30 mM) but much higher than that in mice (10 mM). The rise in salivary Na^+^ concentration also increases the neural response (stimulus dependent), meaning that the Na concentration can be estimated as a function of the neural discharge rate. This capacity allows individuals to ingest the appropriate amount of NaCl to maintain homeostasis, i.e., around 1% NaCl (0.17 M), in the ECF [10,20,26].

Accordingly, the chorda tympani nerve appears to contain two types of fibers that differ in their sensitivity to NaCl; one type responds solely to salty taste (Na^+^ and Li^+^; NaCl specialist, highly specific fibers), while the other responds to a wide range of acid and salty stimuli (NaCl generalist), including Na [3,13,18]. Activity of the former but not the latter is blocked by applying amiloride on the tongue in various animal species, as expected [10].

Chorda tympani nerve fibers with oral saltiness information terminate in a highly specific region of the rostral nucleus of the solitary tract (rNST), especially at its most anterior pole and lateral division [18,27,28].

### 2.2. Sodium Detection and Processing in the Gastrointestinal System

Various studies have demonstrated that the most caudal levels of the digestive system are also prepared for Na detection (visceral sensory signals) [29,30,31,32,33].

Information on the entry of Na can be transmitted from the gastrointestinal system to the CNS in a rapid manner, via neural mechanisms, or more slowly, via the circulatory system (humoral pathway) [29,32,33,34,35,36].

The neural pathway is vagal in nature [37], and Na^+^ detection by subdiaphragmatic vagal afferents is well documented [33,38]. The presence of NaCl-sensitive receptors has been demonstrated in the gastrointestinal mucosa [39,40,41,42] and at hepatic level [43,44]. These receptors appear to be connected to small-diameter fibers, mostly unmyelinated vagal fibers that respond to the presence of hypertonic NaCl with a mean latency of around 10–20 s [39,40,41,42]. The first relay of visceral-vagal information from post-oral segments of the digestive system takes place in caudal segments of the nucleus of the solitary tract (NST), from which it is remitted to other brain structures [27,45,46].

Na absorption into the circulatory system largely takes place in the distal small bowel and the colon [47,48,49]. In these digestive segments, Na^+^ is taken up through absorptive cells of the epithelium, which connect on one side with the intestinal lumen (apical region, brush border) and on the other (basolateral region) with blood and lymphatic capillaries. In the small intestine, these cells are organized into digitiform projections or intestinal villi that significantly augment (around 600-fold) the absorption surface area; however, this organization is less marked towards distal ends of the intestine and is virtually absent at the most distal region of the colon [50].

Na absorption is transcellular in the intestine. In a first step, the ion penetrates from the intestinal lumen into the cytoplasm of the absorptive epithelial cell following electrochemical gradients and strongly assisted by Na-dependent transporters (e.g., Na^+^/glucose and Na^+^/cotransporter amino acids or Na–hydrogen exchangers (NHEs), or by Na reuptake through ENaCs mainly present in the distal colon [50,51]. Once within the epithelial cells, Na^+^ is actively transported towards paracellular spaces by the Na^+^/K^+^ ATPase pump in the basal and lateral membranes of the cells, avoiding retrograde diffusion, from where it enters the bloodstream [47,48,50].

The modality of Na^+^ absorption and the intestinal segment in which it occurs mainly depends on the circumstances. In this way, it appears to occur preferentially in the small intestine after meals, coupled with nutrient absorption, nutrient-dependent Na absorption, Na^+^/glucose, and Na^+^/cotransporter amino acids. In contrast, it largely takes place in the ileum and colon through NHE or ENaCs during interdigestive periods [50,52]. Under Na deficiency conditions, hormones such as aldosterone can increase Na uptake in the intestinal epithelium, stimulating the expression of the transporters that participate in its absorption (especially at colon level). Consequently, there is virtually no Na loss under these circumstances, with <0.5% of the intestinal content of Na being excreted via the feces under normal conditions [47,51,53,54].

Once in the circulatory system, Na is widely distributed throughout the organism, accessing circumventricular brain structures, which lack a blood–brain barrier, via fenestrated capillaries [55,56], and accessing cerebrospinal fluid (CSF) via ion transporters (e.g., ENaC) in epithelial cells of the choroid plexus [57,58,59,60]. Blood and CSF both have a [Na+] concentration of 144 mOsm/L [61].

## 3. Central Nervous System

**Figure 3:**
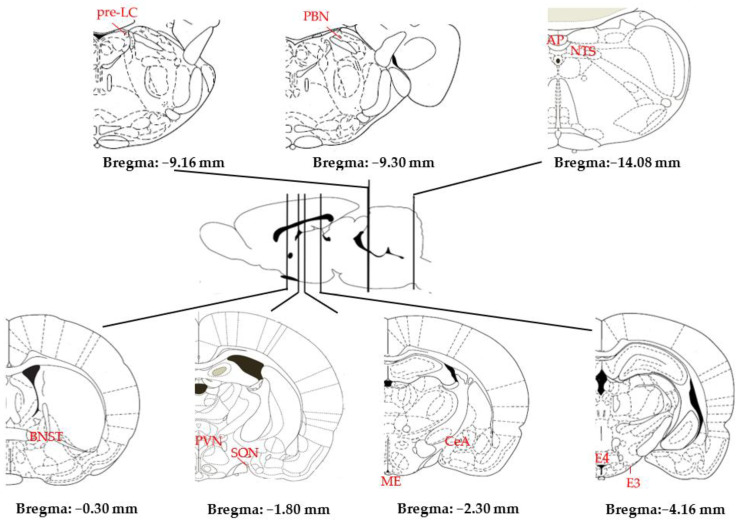
Main structures of the rat brain involved in Na homeostasis (AP: area postrema; BNST: bed nucleus of the stria terminalis; CeA: central amygdala; E3–E4: medial tuberomammillary nuclei; ME: median eminence; NST: nucleus of the solitary tract; PBN: parabrachial nucleus; pre-LC: pre-locus coeruleus; PVN: paraventricular nucleus; SFO: subfornical organ; SON: supraoptic nucleus) (adapted from [63]).

The search for and consumption of water and NaCl depend on neuroanatomical circuits that are far from completely elucidated [8,55,62]. They include numerous interconnected structures widely distributed throughout the CNS, particularly the hypothalamus (HT) and brainstem (see Figure 3). Information from the blood and CSF is processed by circumventricular organs (CVOs) such as the subfornical organ (SFO), which lies in the midline of the third ventricle (IIIV), and the area postrema (AP), which lies in the midline of the fourth ventricle (IVV); information from the chorda tympani nerve originating in the oral cavity is processed by the rNST; and information from the vagus nerve originating in the digestive system is processed by the caudal NST (cNST). Accordingly, these three structures constitute the first information relay in the CNS.

### 3.1. Circumventricular Organs and Nucleus of the Solitary Tract

CVOs are characterized by an extensive network of fenestrated capillaries that permit the entry of Na. These structures also express receptors for hormones that stimulate NaCl intake such as Ang II [64], which is generated by activity of the renin–angiotensin system (RAS) (see Section 4) [8,55,65,66,67].

The SFO is the main CVO for sampling the plasma and CSF. It contains glutamatergic neurons [55,57] with Ang II-sensitive AT1 receptors that stimulate Na intake in Na^+^-depleted and hypovolemic states [8]. In this way, NaCl intake is abolished in AT1a-KO mice or after the intracerebroventricular administration of losartan, an AT1 blocker, under Na-depleted conditions [65]. The activity of these Ang II-sensitive glutamatergic neurons is suppressed in states of cell dehydration by a set of GABAergic interneurons of the SFO [8,55,57]. These GABAergic interneurons are activated by ependymal cells that separate the SFO of IIIV and monitor Na+ ([Na+]) increases in CSF and by astrocytes that monitor [Na+] increases in plasma [57].

The CVO AP is located ventromedial to the fourth ventricle [46,68,69], but its involvement in salt intake is less well understood than that of SFO [58]. The activation of c-Fos in the AP after hypertonic saline injections but not in an Na-deprived state [70] suggests that it forms part of NaCl intake-inhibiting circuits [8]. This inhibitory function may be mediated by serotoninergic neurons that express ENaCs and may detect increases in plasma [Na^+^] to generate a feeling of satiety for this electrolyte [71].

The NST constitutes the first central relay of both gustatory information from the oral cavity and visceral information from the digestive system. The cNST receives visceral-vagal information originating in post-oral segments of the digestive system as well as information from the AP [27,45,46]. The cNST contains neurons sensitive to aldosterone, a lipophilic hormone that crosses the blood–brain barrier. These neurons are activated by chronic dietary Na^+^ deprivation, driving NaCl appetite, and are inactivated after NaCl intake [8]. Conversely, the rNST appears to be involved in the inhibition of NaCl intake. It is the first brain relay for information on oral saltiness carried by the chorda tympani nerve from the tongue and orofacial cavity and possesses a degree of sensorial and viscerotropic organization, i.e., neurons responding to receptors in the anterior tongue are more likely found in the anterior half and those responding to receptors in the posterior oral cavity are in a more caudal position [18,27,46].

**Figure 4:**
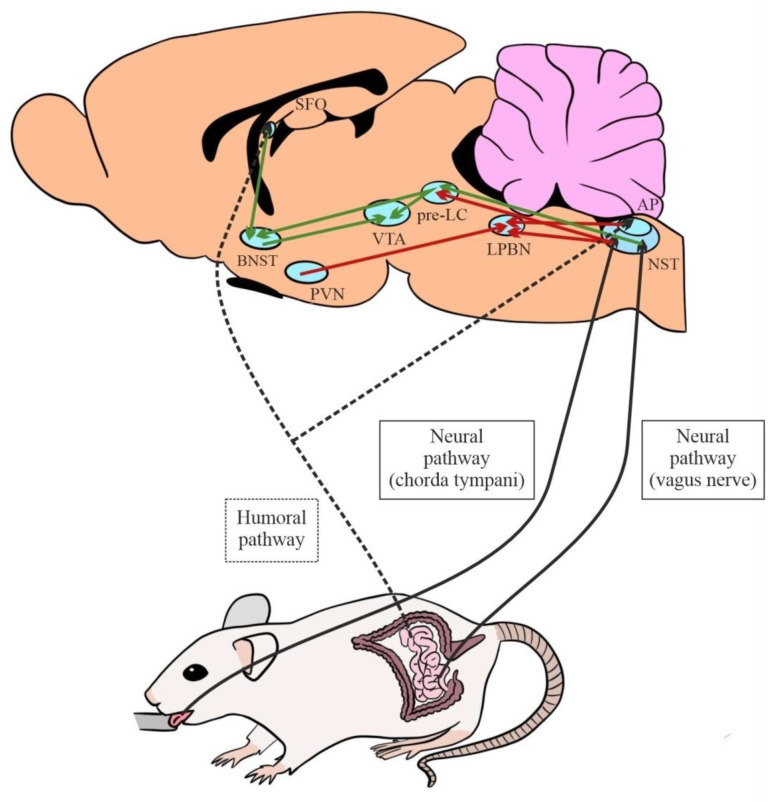
Afferent neural and humoral pathways involved in salt intake and main central excitatory (green) and inhibitory (red) circuits (AP: area postrema; BNST: bed nucleus of the stria terminalis; LPBN: lateral parabrachial nucleus; NST: nucleus of the solitary tract; pre-LC: pre-locus coeruleus; PVN: paraventricular nucleus; SFO: subfornical organ; VTA: ventral tegmental area).

Hence, circuits that stimulate or inhibit NaCl intake originate in the SFO, AP, and NST (Figure 4) (see [8] for a review).

### 3.2. Excitatory and Inhibitory Circuits

SFO glutamatergic efferents activated by Ang II [72] and aldosterone-sensitive cNST neurons [73] project to the ventral region of the bed nucleus of the stria terminalis (vBNST), which therefore appears to integrate both humoral and neural signals [55,69]. This circuit stimulates Na intake, and bilateral electrolytic lesions in the vBNST were found to markedly decrease 0.3*M* NaCl intake by mice under Na-depleted conditions [65]. vBNST neurons project to the ventral tegmental area (VTA) for the regulation of appetitive motivation [74,75,76].

Other efferents that originate in the cNST have proven important to stimulate NaCl intake. One is directed towards the pre-locus coeruleus (pre-LC), where the activation of neurons can stimulate the intake of Na concentrations that are usually aversive [77]. This motivational system can be promoted by projections from the pre-LC towards the vBNST and VTA, among other structures [76]. These pre-LC neurons are inhibited by rNST projections related to the oral detection of Na [8].

An increase in physiological Na levels triggers the activation of inhibitory circuits that limit intake of this electrolyte. In this regard, a decisive role has been described for serotoninergic pathways that project from the AP to the lateral parabrachial nucleus (lPBN) [78]. In this way, pharmacological blockade by the intraparabrachial administration of 5-HT antagonists was found to increase Na consumption [79,80,81,82,83,84,85,86]. Other afferents to the lPBN derive from the rNST. Signals from receptor cells of the tongue that are activated by NaCl intake and project to the rNST were also found to converge in the lNPB, inhibiting its intake [46,87,88]. In addition, lPBN activity and Na intake suppression appear to be enhanced by oxytocinergic afferents from the parvocellular division of the hypothalamic paraventricular nucleus (pPVN) [89]. Inhibition of the lPBN has been related to the intake of highly concentrated (usually aversive) NaCl solutions, associated with endogenous opioid signaling and the central amygdala (see [8]).

### 3.3. Posterior Hypothalamus in Sodium Intake Regulation

The posterior HT is much less explored but no less important for Na homeostasis. Clinical and experimental findings have shown that lesions of mediobasal structures of the posterior HT produce major imbalances in body Na levels and osmolality [90,91,92,93]. Accordingly, Bacic et al. [94] described severe hyponatremia in soldiers with bullet wound injuries to the base of the brain and/or hypothalamic areas adjacent to the IIIV. In some cases, patients are diagnosed with cerebral salt wasting (CSW) syndrome, characterized by hyponatremia and hypovolemia, due to the early natriuresis/diuresis resulting from increased natriuretic peptide activity The most common approach to the restoration of volume and sodium levels in patients with CSW is to administer hypertonic saline solutions and/or salt tablets and limit the free intake of water to minimize the pathological natriuresis, diuresis and polydipsia observed in this disorder [95,96].

These data may suggest a parallelism between CSW patients and animals with lesions in mediobasal hypothalamic structures [97,98,99,100,101,102]. Thus, damage to the median eminence (ME) may interrupt some of the brain systems proposed to be involved in hydromineral regulation (e.g., posterior hypothalamus–hypophyseal axis), causing major derangements in the neuroendocrine control and regulation of water and sodium metabolism [92,103].

Transient activation of the affected neural tissue produced by the application of electric current during surgery [104,105] can generate the increased natriuretic response [92] observed in animals with CSW syndrome [95,96,106]. This mechanism may explain the early excretion of Na (hyponatremia) and hypovolemia developed by ME-lesioned animals during the first few hours post-surgery [92]. It is likely, as in patients with CSW, that the greater preference for hypertonic saline solutions (1.5% NaCl, aversive taste) without a proportional enhancement in water intake observed in ME rats a few hours after the lesion may constitute optimal strategies to restore the volume and composition of extracellular body fluid, of which sodium is one of the main constituents [102].

Other animal studies reported that disruption of the posterior hypothalamus–pituitary axis can cause hypothalamic CDI [92,100,101,103,107]. The altered fluid retention mechanisms observed in these animals can also be accompanied by hypernatremia and/or plasma hyperosmolality [92,108].

Continuing in the clinical setting, findings in patients with schizophrenia included significantly elevated plasma Na levels (hypernatremia) and polydipsia (5–20 L daily), whereas the value of other electrolytes (K^+^, Ca^2+^) remained within normal ranges [109,110,111]. Postmortem studies in these patients revealed a lower bilateral neuronal density (34%) in regions of the posterior HT with respect to individuals without this disorder [112]. Various clinical studies have observed hypernatremia symptoms after injuries to mediobasal hypothalamic areas that affect mechanisms responsible for the conservation of fluids during the chronic phase of central diabetes insipidus (CDI) [97,113].

These different clinical findings are in agreement with the experimental data recorded in our laboratory after electrolytic lesions of tuberomammillary nuclei of the posterior hypothalamus [93].

#### Tuberomammillary System 

The tuberomammillary (TM) system is formed by five nuclei (designated E1–E5) located in the posterior HT on both sides of the mammillary recess [114,115,116,117]. It contains neurons with dendritic processes that extend throughout the ependymal layer of the IIIV, a position that facilitates the response to certain molecules and neuroactive substances in CSF [118,119,120]. Some of its efferents project to the magnocellular division of the paraventricular nucleus (mPVN) and supraoptic nucleus, hypothalamic structures that are responsible for the synthesis of antidiuretic hormone (ADH) and oxytocin (OXY) [7]. Thus, activation of TM cells was found to produce the depolarization of mPVN and SON [121,122,123,124,125,126] and the release of ADH and OXY into the posterior pituitary, from where they accessed the general circulation to modulate kidney function [127,128,129]. Conversely, pharmacologic blockade of TM cells significantly decreased the secretion of ADH [124] and OXY [123,130] in response to osmotic stimuli.

Electrolytic lesions of the medial TM (mTM) nuclei E3 and E4 produce a polydipsic response apparently related to increases in plasmatic Na and difficulties in its excretion [103]. In a study of rats, intraperitoneal administration of hypertonic NaCl produced an exaggerated polydipsic response in these animals [100,101]. Conversely, the administration of OXY, a natriuretic hormone (see Section 4), was found to favor Na excretion and abolish the polydipsia generated by lesion of the E3 nucleus [93]. According to these findings, mTM lesions generate a polydipsic response that may be explained by hydromineral imbalance in the mTM-mPVN/SON–kidney axis, which is involved in hydromineral regulation and is responsible for the secretion of ADH and OXY.

## 4. Physiological Mechanism: Kidney and Sodium Excretion

The third component of Na balance is renal Na excretion (natriuresis), which is dependent on kidney function. Although some Na is lost in feces and sweat, most of the Na in the body is excreted via the kidney. Hence, the kidney plays a central role in achieving a balance between the intake and excretion of NaCl and water, ensuring that ECF volume and cardiovascular homeostasis are maintained at a level consistent with normal physiology [131,132].

**Figure 5:**
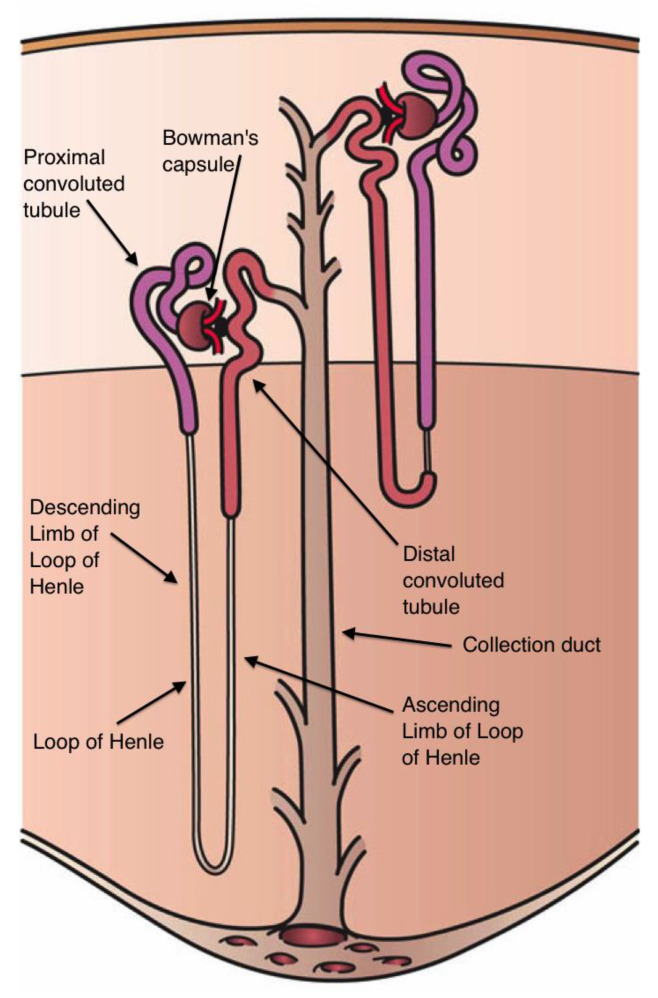
Components of the nephron (From Holly Fischer, Wikipedia commons).

Each human kidney contains more than a million nephrons, its functional units (Figure 5). Blood flows into the kidney through the afferent arteriole, is filtered in a network of small blood vessels, the glomerulus, and is deposited in Bowman‘s capsule, the first element of the nephron. The glomerular filtrate successively passes from Bowman‘s capsule through the proximal tubule, the loop of Henle, with its descending and ascending limb, the distal tubule, and the collecting duct. The walls of eight or ten collecting ducts unite to form a larger single collecting duct that secretes urine. Around 65% of the Na that enters the nephrons is reabsorbed from the renal tubules into interstitial fluid and through peritubular capillaries into the general circulation. The ascending limb of the loop of Henle actively secretes Na into the interstitial space but is impermeable to water, so that the interstitial space between ascending and descending limb is hypertonic (see Figure 5). This hypertonicity creates an osmotic gradient between the fluid within the descending limb and the interstitial space, which removes the water from the descending limb and thereby increases the fluid tonicity within it. Given that the ascending limb is impermeable to water, Na continues to be actively pumped out [132,133].

Natriuresis therefore depends on the ratio between glomerular filtration rate, Na+ tubular reabsorption, and secretion of the fluid containing Na+ into the nephron tubular lumen [132,134].

Na excretion can be modulated by the kidney via renal sympathetic nerves, arterial pressure, and endocrine factors such as the RAS, aldosterone, cardiac natriuretic peptides, ADH, and OXY [4,131,135].

### 4.1. Renin–Angiotensin System

At the end of the 19th century, Tigerstedt and Bergmann [136] demonstrated that injections of renal extracts elevate the blood pressure of normotensive animals. This was the first evidence that the kidney, long acknowledged to be an exocrine organ, might also possess important endocrine functions in the regulation of cardiovascular and body fluid homeostasis. Tigerstedt and Bergmann designated the biologically active substance isolated from the kidney as “renin”. This enzyme is synthesized by specialized granular cells (“juxtaglomerular cells”) of the afferent arteriole of each glomerulus. Renin converts plasma angiotensinogen [137,138,139] into angiotensin I, which is in turn transformed by angiotensin-converting enzyme into Ang II [140].

The main role of the RAS is to maintain the volume of ECF in hypovolemic situations, sustaining arterial pressure and exerting direct and indirect effects on renal Na excretion [141,142,143]. Indirect effects of the RAS include a decrease in peritubular capillary pressure (efferent arteriolar constriction) and increase in Na transport. Direct effects include the action of Ang II on AT1 receptors in the kidney to mediate hemodynamic and tubular function, including afferent and efferent arteriolar vasoconstriction and the enhancement of Na and fluid reabsorption [142,144]. Activation of AT1 receptors increases activities of the proximal tubule apical NHE and basolateral Na-bicarbonate cotransporter. Ang II also enhances activities of the Na-chloride cotransporter in the distal tubule and the ENaC in the collecting duct [145,146,147,148]. Hence, Ang II directly increases the receptor-mediated reabsorption of NaCl (antinatriuresis) and water (antidiuresis) in multiple nephron segments in order to conserve ECF volume [142,149]. In addition, as mentioned above, Ang II acts on AT1 receptors in the SFO to stimulate Na intake [8].

Ang II also stimulates the secretion of aldosterone and the sympathetic nervous system and elevates the blood pressure [150,151].

### 4.2. Aldosterone

In 1936, Curt Richter reported that Na excretion is reduced by the administration of adrenal cortical extracts, whereas extreme Na loss results from adrenalectomy [152]. This antinatriuretic effect is mediated by the action of aldosterone [153], a mineralocorticoid synthesized from zona glomerulosa cells of the adrenal cortex [154]. Its synthesis depends on the hypothalamic–pituitary–adrenal neurosecretory axis (hypothalamic corticotropin-releasing hormone and anterior pituitary adrenocorticotropin hormone), while the strongest stimuli triggering its secretion are hyperkalemia and Ang II [155,156,157,158]. In fact, zona glomerulosa cells are more responsive to an increase in plasma potassium levels (hyperkalemia), while aldosterone accounts for only a small percentage of the Na reabsorbed in the nephron [159,160].

After its release, aldosterone acts on mineralocorticoid receptors located in the distal tubule and collecting duct of the nephron to increase apical Na influx, Na reabsorption, and potassium excretion through Na/ENaCs and potassium channels by Na+/K+ ATPase [161,162,163,164,165,166]. The blood pressure is also increased by these changes [167].

### 4.3. Pressure Natriuresis

In 1854 and 1949, respectively, Goll and Selkurt et al. observed that acute pressure natriuresis can occur without major changes in renal blood flow or glomerular filtration rate [131,165]. The nephron sites most sensitive to renal perfusion pressure changes are the proximal tubules and/or descending limb of the loop of Henle in deep nephrons [168,169].

Increases in renal perfusion pressure [150,170] appear to reduce Ang II levels and increase nitric oxide (NO) production, directly inhibiting tubular Na reabsorption. Although the action mechanisms of NO are not well known, the presence of various isoforms of its synthesis enzyme, NO synthase, has been identified in endothelial cells of the renal vasculature and glomerular capillaries, renal sympathetic nerves, renal tubules, macula densa, and medullary regions [171]. Conversely, a reduction in renal perfusion pressure produces an increase in tubular Na reabsorption and a decrease in Na excretion [150].

Acute increases in arterial pressure restore Na excretion to normal values when the amount of Na excreted via pressure natriuresis is greater than its intake [150,167]. A rise in renal perfusion pressure depresses proximal tubular fluid reabsorption, increases fluid excretion, and reduces ECF volume, producing a short-term reduction in blood pressure to its baseline value [131]. Consequently, a balance is achieved between pressure natriuresis and ECF volume [150,167].

### 4.4. Sympathetic Nervous System

Muller and Barajas described kidney innervation by sympathetic post-ganglionic fibers in 1972. Norepinephrine (NE)-containing renal sympathetic nerve terminals are in direct contact with all renal tubular segments and with juxtaglomerular granular cells [172].

Renal sympatho-excitation produces vasoconstriction, antinatriuresis, and antidiuresis. Conversely, renal sympatho-inhibition results in vasorelaxation, natriuresis, and diuresis [171,172,173,174]. Sympathetic NE acts on the kidney at the following primary sites: renal vasculature, causing vasoconstriction; epithelial cells of proximal tubules, ascending thick limb of the loop of Henle and distal tubules, stimulating water and Na reabsorption; and the juxtaglomerular apparatus, producing the release of renin [131,171,172,174,175]. These effects result from the action of NE on G proteins coupled kidney adrenoceptors. When stimulated, β1-adrenoceptors of juxtaglomerular granular cells cause the release of renin [176], while α1-adrenoceptors of vascular smooth muscle cells produce a contraction that reduces the renal blood flow and/or glomerular filtration rate [177], and α1-adrenoceptors in nephron tubules enhance the cAMP accumulation induced by ADH to promote Na and water reabsorption, acting via the Na+/K+ ATPase pump [171,178]. Conversely, activation of tubular α2-adrenoceptors reverses ADH-induced Na and water retention [179]. Furthermore, presynaptic sympathetic α2-adrenoceptors decrease the amount of NE released by subsequent action potentials [171].

The sympathetic nervous system interacts with the RAS and with cardiac natriuretic peptides [180,181]. Ang II was found to enhance NE neurotransmission via AT1 receptors [171,182] and the blockade of AT1 receptors blunted renal nerve-mediated reductions in urinary Na excretion and flow rates [183]. It was also reported that atrial natriuretic peptide (ANP) can negatively modulate noradrenergic neurotransmission and reduce NE release from the rat adrenal medulla [184].

### 4.5. Cardiac Natriuretic Peptides

In 1981, De Bold et al. observed natriuresis and diuresis after the injection of an atrial homogenate [185], later identified as ANP, revealing the heart to be an endocrine organ [186,187,188,189]. ANP is also found in the brain, and perikarya containing elevated ANP levels were observed in the preoptic-hypothalamic area and magnocellular hypothalamic nuclei of rats [184]. Cardiac tissue also produces brain natriuretic peptide (BNP), initially identified in the brain [190,191]. ANP and BNP are secreted in response to cardiac wall stretching and extracellular volume expansion [192], while hypovolemia reduces the release of these natriuretic peptides [193,194].

In the kidney, ANP and BNP act on natriuretic peptide receptor A (NPR-A) in juxtaglomerular cells, proximal tubule, thin and thick ascending loop of Henle, and collecting duct [195,196,197,198,199]. ANP and BNP generate diuresis and natriuresis by inducing vasodilation of the afferent glomerular arteriole and vasoconstriction of the efferent glomerular arteriole, increasing the glomerular filtration rate [200]. The NPR-A also modulates activity of the cGMP-gated cation channel that mediates electrogenic Na absorption [195,201], inhibiting water and Na reabsorption [202,203,204,205,206].

As expected, natriuretic peptides have antagonistic effects on the RAS, given that ANP decreases renin secretion from juxtaglomerular cells, reduces Ang II, blocks aldosterone synthesis and release, and suppresses ADH secretion [207,208,209,210].

### 4.6. Antidiuretic Hormone

The role of the pituitary gland in body fluid homeostasis was first noted in the early 1900s after observing that the exogenous administration of a pituitary extract reduced the urine flow [211].

The hormone responsible for these effects is ADH, which is synthesized in the somata of magnocellular neurons of hypothalamic paraventricular (mPVN), supraoptic (SON), and accessory neurosecretory nuclei. Their axons pass through the median eminence (ME), forming the neurohypophyseal stalk, and terminate in the posterior pituitary, entering the circulation thereafter [7,212].

Increases in ADH release are more sensitive in response to a rise in plasma Na concentration than to hypovolemia or hypotension [213,214]. It acts via two types of receptors in the kidney: receptor V2, which mediates both antidiuresis and antinatriuresis; and receptor V1a, which mediates natriuretic effects. ADH increases water reabsorption by acting on V2 receptors in the collecting duct of the nephron [193], promoting the production and expression of water aquaporin 2 (AQP2) channels that permit water reabsorption [215]. Consequently, tubular fluid traversing this segment of the nephron equilibrates osmotically with the hyperosmotic interstitium [134,216]. In this way, the kidneys can process urine that has a concentration higher than serum osmolality [133], with the collecting ducts playing a key role in both the dilution and concentration of urine (excretion and conservation of water). Conversely, this region is impermeable to water under conditions of normal osmotic equilibrium in the absence of ADH, resulting in the continuing dilution of luminal fluid and excretion of diluted urine [135]. V2 receptors are also involved in the action of ADH to reduce renal Na excretion [217]. ADH Na reabsorption is mediated by ENaCs in the thick ascending limb [147,218], and ADH increases the number of ENaCs [219] and the apical Na^+^ reabsorption [220]. This action also produces a reduction in water excretion [214,221,222,223,224]. In this way, the activation of V2 favors antidiuresis associated with antinatriuresis in the presence of hypovolemia [225,226,227]. On the other hand, when ADH is introduced at higher doses, its activation of V1a receptors in connecting tubules and collecting ducts inhibits renal Na reabsorption and favors natriuresis [134,217,228]. These findings suggest that V1a and V2 receptors are activated in states of hypernatremia, promoting natriuretic and antidiuretic effects [134,225,226,227].

ADH is the only hormone traditionally related to diabetes insipidus (DI), characterized primarily by the excretion of a large volume of diluted, “tasteless” urine and secondarily by excessive water intake (polydipsia) and the presence of hypernatremia [229,230,231]. Two main DI types can be distinguished: central, neurogenic, neurohypophyseal, or hypothalamic DI (CDI), associated with deficient secretion of ADH, and nephrogenic DI, characterized by renal insensitivity to ADH.

CDI has been treated with ADH analogs [232], although nephrectomy does not prevent the polyuric response [233], indicating the implication of additional factors. In this regard, most alterations caused by CDI affect the secretion of both ADH and OXY [7,212,232,234,235,236], suggesting the possible involvement of OXY.

### 4.7. Oxytocin

Besides the aforementioned vasopressinergic neurons, the magnocellular neurosecretory system also contains cells responsible for OXY synthesis [237]. As in the case of ADH, OXY is generated in response to increased plasmatic osmolality (hyperosmolality and hypernatremia) and hypovolemia [7,212]. OXY is especially involved in the excretion of body Na [229,238,239,240,241,242], even at physiological plasma concentrations [243]. This physiological secretion appears to be triggered directly by increases in glomerular filtration rate [244] and reductions in tubular Na reabsorption [245] and indirectly by the cardiac secretion of ANP [246]. In addition, ADH and OXY have been reported to exert synergic natriuretic effects [247,248].

Urine excretion, water intake [229], and Na appetite [240] are increased by the natriuretic effects of OXY in food-deprived rats and in rats fed ad libitum with a low-sodium diet. Hence, OXY administration and low-sodium diets can produce negative Na and water balances [243] that can be counteracted by consuming Na in the diet or by hypertonic NaCl administration [239,240].

Over the past few decades, the role of OXY and Na balance disorders has been investigated in patients and animal models of CDI [212,234]. As a result, treatments of this disease have started to involve not only ADH but also OXY administration and food-deprivation and low sodium diets, both in animals [92,101,242,249,250,251] and humans [252,253,254]. The aim is to exert natriuretic and antidiuretic effects, potentially reducing the characteristic hypernatremia in CDI [92,253,255,256].

As already noted above, intraperitoneal administration of OXY was found to favor Na excretion and reduce the polydipsic response to the lesion of medial TM nuclei [93].

### 4.8. Salt Sensitivity and Hypertension

As already noted, kidney function can affect not only sodium excretion but also blood pressure, and some individuals respond to high salt intake with an increase in blood pressure [257]. In this context, salt sensitivity refers to changes in blood pressure levels parallel to changes in salt intake [258,259].

The kidney responds to increases in salt intake by activating natriuretic mechanisms (pressure natriuresis and cardiac natriuretic peptides) and inhibiting antinatriuretic ones (RAS-aldosterone and sympathetic nervous system). The former mechanisms are not sufficiently activated in salt-sensitive individuals, and the latter are not effectively suppressed [258,260]. Salt intake is of major clinical relevance in salt-sensitive individuals as a key element in the pathophysiology of hypertension [257]. The gut microbiota has also been related to salt sensitivity and hypertension development [261,262].

The trillions of bacteria, viruses, protozoa, archaea. and fungi that reside on the mucosal surface of the human gastrointestinal tract (gut microbiota) have proven crucial to maintain the homeostasis, metabolism, and immunity of the host [263,264]. The involvement of the gut microbiome in these functions would explain why its alteration (dysbiosis) is associated with numerous diseases, including hypertension [264,265].

The diet is an important determinant of the composition of the gut microbiota [264]. Recent findings demonstrated that a high salt intake is associated with major changes in the gut microbiome (modifications in bacterial taxa, dysbiosis) that are in turn related to salt sensitivity in hypertension [262,265]. A significant increase in Firmicutes, with an overall increase in the Firmicutes/Bacteroidetes ratio, has been described in humans with hypertension [266], leading to the proposal of the microbiota as therapeutic target in this disease. In this way, various prebiotics, probiotics, and postbiotics have been proposed as potential treatments due their antihypertensive effects [265].

The mechanisms by which gut microbiota disorders may cause hypertension are not fully elucidated, although the production of different circulating metabolites has been related to renal sodium regulation, vascular function, and immune processes [265].

## 5. Concluding Remarks

Na is essential for life, and its regulation depends on the balance between its intake (behavioral component) and its loss, mainly controlled by the kidney (physiological component). Numerous brain structures are involved in monitoring Na levels and forming circuits to stimulate or inhibit its intake in accordance with homeostatic and hedonic factors.

Many of the chemical substances involved in the above processes act in a coordinated manner. They are responsible for intrabrain effects as neurotransmitters that regulate the behavioral component and for hormonal effects on the oral cavity and gastrointestinal tract, modulating Na hunger, and on the kidney, regulating the physiological component (natriuresis). In Na-deficient animals, aldosterone enhances the gustatory response to Na by acting on taste cells [22] and increases its uptake in the intestinal epithelium [47,51,53,54]. In the CNS, aldosterone stimulates Na intake by modulating the activity of the NST [73]. Finally, it increases Na reabsorption by acting on nephrons [161,162,163,164,165,166]. Ang II also increases the gustatory response to Na in Na-deficient animals [22], activates glutamatergic neurons of the SFO that stimulate Na intake [8], and has renal effects that enhance Na reabsorption [142,147]. Accordingly, aldosterone and Ang II protect the organism against hypovolemic and hyponatremic states, whereas other chemical substances protect the organism against hypervolemia and hypernatremia. This is the case of OXY, which acts on the lPBN to inhibit Na intake [89] and acts on the kidney to stimulate Na excretion [243].

Besides Na homeostasis, these and other brain structures and chemical messengers participate in the regulation of other closely related processes (e.g., hydric homeostasis and cardiovascular regulation), hampering differentiation between direct and indirect effects. In this way, variations in body Na levels affect not only the intake and excretion of this solute but also the intake and excretion of fluids, as observed in Figure 1.

## Data Availability

Not applicable.
